# Plastic Burning Impacts on Atmospheric Fine Particulate Matter at Urban and Rural Sites in the USA and Bangladesh

**DOI:** 10.1021/acsenvironau.1c00054

**Published:** 2022-06-09

**Authors:** Md. Robiul Islam, Josie Welker, Abdus Salam, Elizabeth A. Stone

**Affiliations:** † Department of Chemistry, University of Iowa, Iowa City, Iowa 52242, United States; ‡ Department of Chemistry, University of Dhaka, Dhaka 1000, Bangladesh; § Department of Chemical and Biochemical Engineering, University of Iowa, Iowa City, Iowa 52242, United States

**Keywords:** atmospheric aerosol, urban aerosol, air quality, trash burning, garbage burning, molecular markers, triphenylbenzene

## Abstract

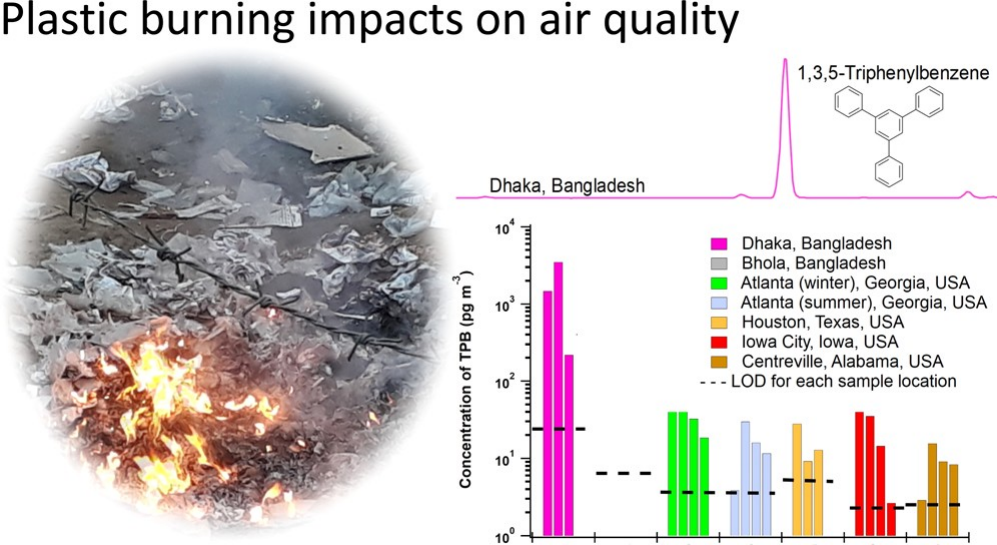

To better understand the impact of plastic burning on atmospheric fine particulate matter (PM_2.5_), we evaluated two methods for the quantification of 1,3,5-triphenylbenzene (TPB), a molecular tracer of plastic burning. Compared to traditional solvent-extraction gas chromatography mass spectrometry (GCMS) techniques, thermal-desorption (TD) GCMS provided higher throughput, lower limits of detection, more precise spike recoveries, a wider linear quantification range, and reduced solvent use. This method enabled quantification of TPB in fine particulate matter (PM_2.5_) samples collected at rural and urban sites in the USA and Bangladesh. These analyses demonstrated a measurable impact of plastic burning at 5 of the 6 study locations, with the largest absolute and relative TPB concentrations occurring in Dhaka, Bangladesh, where plastic burning is expected to be a significant source of PM_2.5_. Background-level contributions of plastic burning in the USA were estimated to be 0.004–0.03 μg m^–3^ of PM_2.5_ mass. Across the four sites in the USA, the lower estimate of plastic burning contributions to PM_2.5_ ranged 0.04–0.8%, while the median estimate ranged 0.3–3% (save for Atlanta, Georgia, in the wintertime at 2–7%). The results demonstrate a consistent presence of plastic burning emissions in ambient PM_2.5_ across urban and rural sites in the USA, with a relatively small impact in comparison to other anthropogenic combustion sources in most cases. Much higher TPB concentrations were observed in Dhaka, with estimated plastic burning impacts on PM_2.5_ ranging from a lower estimate of 0.3–1.8 μg m^–3^ (0.6–2% of PM_2.5_) and the median estimate ranging 2–35 μg m^–3^ (5–15% of PM_2.5_). The methodological advances and new measurements presented herein help to assess the air quality impacts of burning plastic more broadly.

## 1 Introduction

Of the 2.4 billion tons of solid waste generated globally each year, approximately 26% is burned residentially and 15% is burned at dump sites.^1^ Regulations surrounding the handling, transport, storage, and disposal of solid waste vary across the globe, from landfill and recycling to incidental or intentional combustion.^2^ In the USA, an estimated 1.3% of 226.9 million metric tons of domestic solid waste generated annually is burned residentially or at dump sites, compared to 60% of 23.7 million tons in Bangladesh.^1^ The higher rates of combustion in Bangladesh are associated with the open burning of waste along roadways or at dump sites. Waste burning is estimated to be a major global source of air pollutants relative to known anthropogenic sources, especially for carbon monoxide, particulate matter (PM), mercury, hydrochloric acid, and polycyclic aromatic hydrocarbons (PAHs).^1^

The burning of waste emits large quantities of PM, with emission factors typically ranging from 5 to 50 mg PM per kilogram of fuel burned (mg kg^–1^) and the quantity and chemical composition of emissions varying with fuel composition and combustion conditions.^2−10^ PM emitted from waste burning is toxic^3,12^ and contains organic and elemental carbon, chloride, polycyclic aromatic hydrocarbons (PAHs), phthalates, bisphenol A, and toxic metals (e.g., Sb, Cu, Zn, Pb, V, As).^2,4,7−9,12,15−17^ Polychlorinated dibenzodioxins and polychlorinated dibenzofurans are emitted from burning chlorine containing plastic and are highly toxic, teratogenic, mutagenic, and carcinogenic.^3,7,18^ Waste burning also emits gas-phase hydrochloric acid, nitrogen dioxide, formaldehyde, and other volatile organic compounds.^19^

Although studies on waste burning impacts on air quality are rare, it has been estimated to have a large impact in some urban airsheds. For example, the impact of garbage burning in the Mexico City Metropolitan Area where garbage burning contributes to fine particles less than 2.5 μm (PM_2.5_) was estimated to be 28,^8^ 1–15,^20^ and 3–30%,^21^ by differing measurement and/or modeling techniques. Spatially resolved data indicate that the distribution of PM from garbage burning is highly variable across Mexico City, with the greatest relative impact occurring in highly populated suburban locations.^20^ The open burning of garbage was estimated to contribute 18% of PM_2.5_ organic carbon in a suburb of Kathmandu, Nepal, placing it alongside biomass burning and fossil fuel use as a major source of PM_2.5_.^22^ Garbage burning has also been estimated to contribute 4.7% of PM_2.5_ organic carbon in Lumbini, Nepal.^23^ Plastic burning specifically has been estimated to contribute 13.4% of PM_2.5_ in Delhi, India,^24^ and contribute 6.8% of PM_2.5_ in Nanjing, China.^25^ The substantial impact of waste burning on air quality has been supported by modeling in South Asia.^4,26^

As a means of tracking plastic burning in the atmosphere, several chemical tracers have been proposed: metals that are uniquely enriched in solid waste burning emissions (e.g., Sb, As, Sn, and/or Cd) and organic compounds, such as triphenylbenzene, phthalates, or terephthalic acid.^2,3,15,27^ Among the possible tracers, this study focuses on 1,3,5-triphenylbenzene (TPB), which is produced from burning plastic and has been recommended as a tracer of PM emitted from the combustion of plastics and landfill waste.^2,10,27^ It has not been detected in other types of combustion emissions, including fossil fuels and biomass, indicating that it is unique to plastic combustion.^9,27^ Laboratory studies involving various plastic materials indicate that TPB is particularly enhanced in emissions from plastics with an aromatic ring in their structure, such as polystyrene and polyethylene terephthalate.^10^ TPB has been detected in atmospheric PM samples collected in Santiago, Chile;^2,28^ Mexico City, Mexico;^29^ Taizhou, China;^30^ Okinawa, Japan;^31^ Kathmandu, Nepal;^22^ Lumbini, Nepal;^23^ Chennai, India;^32^ Bucharest, Romania;^10^ Wadowice, Poland;^33^ and other sites reviewed by Simoneit et al.^27^ Measurements of TPB in the USA are infrequent; it was not detected in PM samples from Los Angeles, California and Corvallis, Oregon^2^ and in only 26% of samples collected at a remote mountain top site on Mt. Bachelor, Oregon.^34^

The objectives of this study are threefold. First, we demonstrate that gas chromatography (GC) mass spectrometry (MS) may be used to quantify TPB, following solvent extraction of substrates containing PM or thermal desorption (TD) by direct sample introduction developed by Yu and co-workers for PAH and other molecular markers.^35−37^ Second, we apply this TD-GCMS method to ambient fine particulate matter (PM_2.5_) collected at four locations in the USA and two locations in Bangladesh. These measurements add to sparse measurements of this compound in ambient PM in each country. Third, we roughly estimate the potential impact of plastic burning on ambient PM_2.5_ using emissions data from prior plastic and waste burning studies.^2,9,10^ Together, these objectives advance the use of TPB as a tracer for plastic burning in ambient PM and provide new insight to the air quality impacts of this source.

## 2 Materials and methods

### 2.1 Sample collection

PM_2.5_ samples were collected at four sites in the USA and two sites in Bangladesh as part of prior studies and were reanalyzed in this study for TPB. At the four sites in the USA, PM_2.5_ samples were collected onto pre-baked quartz fiber filters (QFFs) using a medium-volume PM_2.5_ sampler (URG Corp.) at 90 L min^–1^. Field blanks were collected at a rate of one per five samples. Additional details of the study site, sample collection, and co-located measurements are provided in preceding articles for each respective site: Iowa City, Iowa (24 h daily samples in 2015);^38^ Atlanta, Georgia (24 h daily samples);^39^ Houston, Texas (12 h day/night samples);^40^ and Centreville, Alabama (12 h day/night samples).^41^ Ten additional PM_2.5_ samples were collected at the Iowa City site from October 16, 2020, to November 15, 2020, over 72 h intervals following the methods described previously.^38^ At the Bhola^42^ and Dhaka^43^ sites in Bangladesh, PM_2.5_ samples were collected on QFF with a low volume sampler (Envirotech APM 550, Envirotech Instrument Pvt. Ltd.) operating at 16.7 L min^–1^. For comparison of solvent-extraction and TD-GCMS methods, select PM_2.5_ samples from three sites in Nepal (Kathmandu, Lalitpur, and Lumbini^23^) that had been previously analyzed for TPB by solvent-extraction GCMS were reanalyzed using TD-GCMS, with sampling methods, TPB measurements, and source impacts on PM_2.5_ are discussed elsewhere.^23^ Briefly, samples in Nepal were collected by a medium-volume sampler with eight channels (ABC-3000, URG) each operating at approximately 8 L min^–1^ onto QFF or Teflon filters. Co-located measurements of PM_2.5_ mass were determined gravimetrically or by a tapered element oscillating microbalance (TEOM, Thermo Fisher) in the case of Centreville, AL.^44^ Organic carbon (OC) and elemental carbon (EC) determined by thermal-optical methods^45^ are reported when data are available.

### 2.2 Measurement of TPB by Solvent-Extraction GCMS

All glassware (Pyrex) was baked at 500 °C for 5 h and 30 min before use. Prior to extraction, all substrates were spiked with 100 μL of an internal standard solution containing benzo(a)anthracene-*d*_12_ at 500 pg μL^–1^ (Cambridge Isotope Laboratory Inc., 98.0%) using a glass microsyringe (100 μL, Hamilton). After drying, substrates were extracted using acetonitrile (Fisher Scientific, 99.9%) by ultrasonication (Branson 5510). The extracted solution was filtered using a 0.2 μm poly(tetrafluoroethylene) (PTFE) filter (Whatman, GE Health Care Life Sciences) and concentrated under high-purity nitrogen (>99.999%, PRAXAIR Inc.) with gentle heating (Caliper Life Sciences, Turbo Vap LV Evaporator; Thermo Scientific, Reacti-Vap Evaporator) to a final volume of 100 μL, as described by Al-Naiema et al.^46^ The concentrated solution was then analyzed by gas chromatography (GC; Agilent Technologies 7890A) coupled to mass spectrometry (MS; Agilent Technologies 5975C) using a temperature program described in Stone et al.^47^ The GC separation utilized a DB-5 capillary column (5% diphenyl/95% dimethylsiloxane; 30 m × 0.25 mm × 0.25 μm; Agilent; Santa Clara, CA). The MS was operated in scan mode from *m*/*z* 50 to 1000 at an ion source temperature of 230 °C and 70 eV for the electron impact ionization mode. Instrument operating conditions are summarized in Table S1.

### 2.3 Measurement of TPB by TD-GCMS by Direct Sample Introduction

For TD-GCMS analysis, QFF subsamples were analyzed by directly introducing the sample to the GC inlet. The subsample was typically cut by a stainless-steel filter punch (1.0 cm^2^, Sunset Laboratory Inc.) or standardized circular cork punch on a surface of a pre-baked aluminum foil. A 3 μL aliquot of internal standard solution (benzo(a)anthracene-*d*_12_ at 500 pg μL^–1^ in toluene) was added onto the filter strip using a glass microsyringe (5 μL, Hamilton) and allowed to evaporate. A 1.0 cm^2^ filter punch was typically cut into four roughly equal strips with a razor blade that were loaded into a clean (pre-baked at 500 °C for 10 h) splitless GC inlet liner (5190-2271, Agilent) using pre-cleaned stainless-steel tweezers.

The sample and inlet liner were loaded into the GC inlet and heated to 50 °C. The temperature programs for the GC inlet and column along with the thermal-desorption steps followed prior studies^35−37^ and are summarized in Figure S1. Optimization of the inlet temperature and desorption time are shown in Figure S2. The injector was first set in the splitless mode in the GC temperature program and switched to the split mode after 13 min. The carrier gas was ultra-high-purity (99.9999%) helium (PRAXAIR Inc.) held at a constant flow of 1.0 mL min^–1^. The GC column and MS parameters are summarized in Table S1 and mass spectra are shown in Figure S3.

### 2.4 Calibration, Quality Control, and Performance Metrics

Calibration standards of TPB (TCI America, >99.0%) were prepared in distilled toluene (Sigma-Aldrich, 99.8%) and contained isotopically labeled benzo(a)anthracene-*d*_12_ as an internal standard. The linear range of calibration was determined by subsequently injecting the calibration solutions from low to high TPB concentrations. To assess the extraction recovery by solvent extraction and TD-GCMS, six QFF spiked with known concentrations of TPB (200 pg μL^–1^ in toluene) were prepared by solvent extraction and thermal desorption and analyzed by GCMS. The spike recovery was calculated as the ratio of the recovered spike concentration to the spiked concentration. TPB was not detected in laboratory blanks (*n* = 2) or field blanks (*n* = 8), making blank subtraction unnecessary. Additionally, 15 atmospheric PM_2.5_ samples from Nepal and 3 field blanks were analyzed by both methods for comparison across the methods. Because TPB was not detected in field or laboratory blanks, the limit of detection (LOD) was calculated from the sum of the calibration curve intercept and three times the standard error of the estimated peak area ratio following Ho and Yu.^36^

## 3 Results and Discussion

### 3.1 Comparison of Solvent-Extraction and Thermal-Desorption GCMS for the Quantification of TPB

Both methods of sample preparation enabled the quantification of TPB over a range of concentrations (Table 1), with acceptable spike recoveries (within ±20% for each of the six spiked samples analyzed by each method). The TD approach enabled quantification over a wider linear range, including more precise measurements at lower concentrations as indicated by its lower limit of detection. The precision of spike recoveries is also improved for TD over solvent extraction. Because the GCMS instrument detection limit for TPB applies to both methods of sample preparation, the detectability of TPB thus depends upon the amount of TPB injected into the instrument. This depends upon the concentration of TPB in the atmosphere and the equivalent volume of the air sample undergoing analysis. To maximize detection of TPB, greater amounts of substrates and/or more heavily loaded substrates may be analyzed. Additionally, a quadrupole mass spectrometer could be operated in single-ion-monitoring (SIM) mode to increase sensitivity in the measurement of TPB. Compared to the solvent-extraction method, the TD-GCMS method provides higher throughput as indicated by lower analysis time per sample. TD-GCMS also minimized the use of organic solvent, requiring small amounts for standard preparation and solvent rinsing.

**Table 1:** Comparison of Method Performance Metrics between Liquid Injection Used in Organic Solvent Extraction and Thermal Desorption (by Direct Sample Introduction) GCMS Analysis of TPB

performance metric	solvent extraction	thermal desorption
analysis time per sample (h)	5	1.5
solvent used per sample (mL)	50	<5
linear calibration range (pg)	40–800	17–10 000
limit of detection (pg)	38	16
correlation coefficient (*R*^2^)	>0.999	>0.999
spike recovery (%), *n* = 6	80–106	99–106

**Figure 1:**
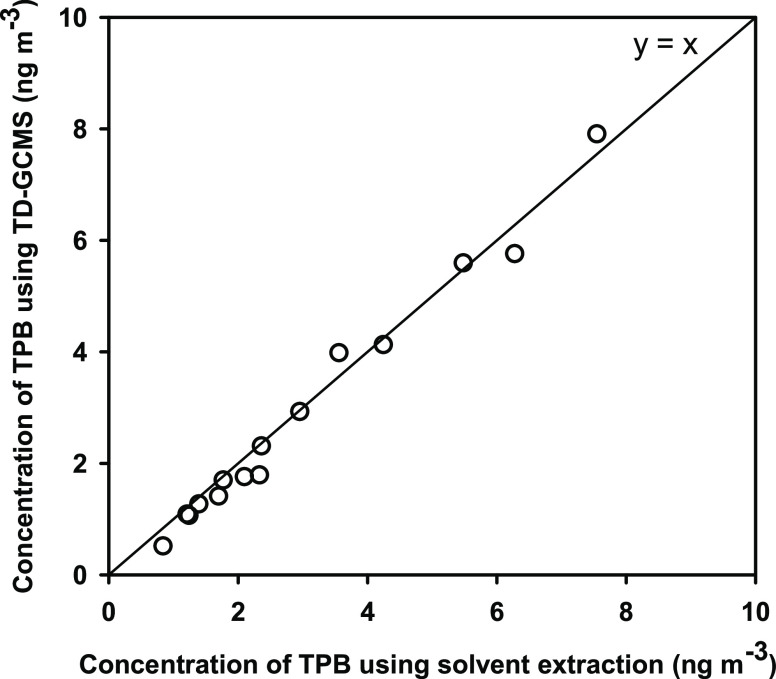
Comparison of TPB concentrations measured using solvent extraction and thermal-desorption GCMS for ambient PM_2.5_ Nepal samples from Lumbini, Kathmandu, and Lalitpur.

Solvent-extraction and TD-GCMS methods were applied to quantify TPB in 15 atmospheric PM_2.5_ samples from Nepal (Figure 1). The concentrations of TPB obtained by these two methods agreed very well, with least-squares linear regression yielding a slope of 0.99 ± 0.02 and a squared correlation coefficient (*R*^2^) of 0.994. These results indicated that TD-GCMS was able to reproduce TPB measurements made by traditional solvent-extraction methods. The successful quantification of TPB by solvent-extraction and TD approaches demonstrates that commonly used methods for quantification of molecular markers in PM_2.5_ can be readily adapted to include TPB.

### 3.2 Detection and Quantification of TPB in ambient PM_2.5_

**Figure 2:**
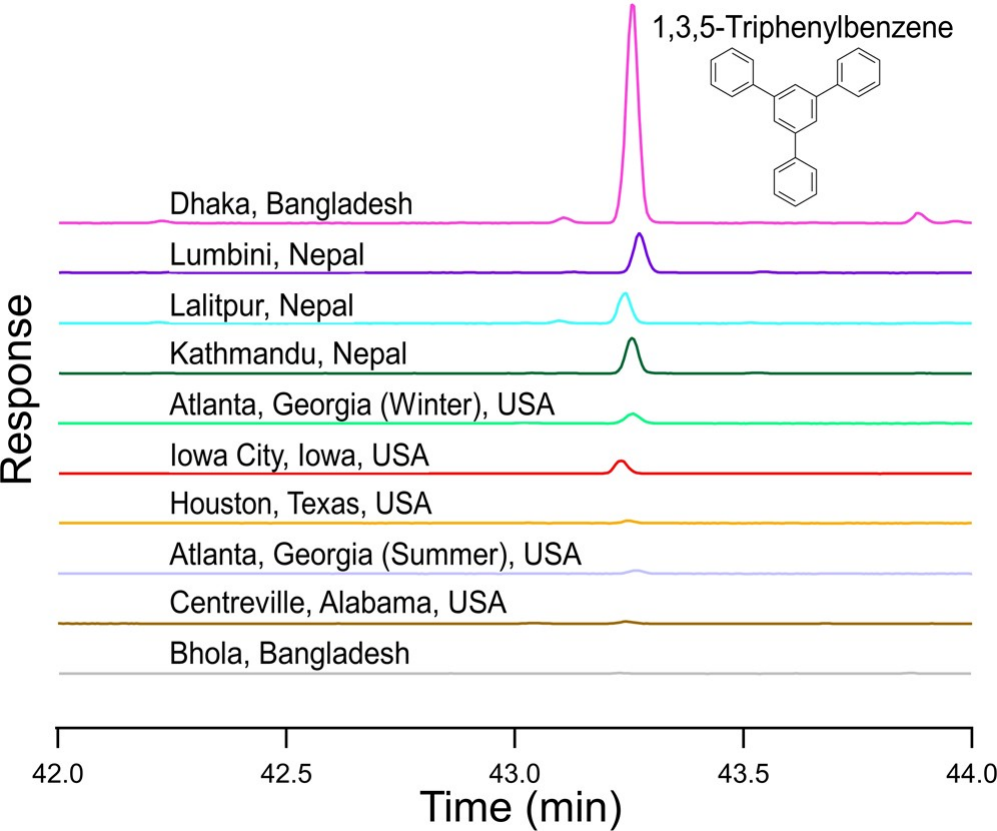
Extracted ion chromatograms for the molecular ion of TPB (*m*/*z* 306); one chromatogram is shown per site and/or season. The retention time for TPB on the DB-5 column is approximately 43.2 min and varies slightly across samples.

TPB was detected at five of six study sites using TD-GCMS (Table 2) by its molecular ion at *m*/*z* 306 at a retention time of 43.2 min (Figure 2), in agreement with the TPB standard. Qualifying ions at *m*/*z* 289 and 228 had lower relative abundance (Figure S3) and were detected in most samples excluding those from Atlanta and the two samples with the lowest concentrations in Houston. For TPB to be reported, its concentration exceeded the limit of detection (Table 1). The only site at which TPB was not detected was the Island of the Bay of Bengal (Bhola) in Bangladesh, which is a remote coastal site. TPB was also not detected in any field blank samples.

**Table 2:** Summary Locations and Dates of Sample Collection and Measurements of PM_2.5_ Mass, Organic Carbon, Elemental Carbon, and 1,3,5-triphenylbenzene (TPB)

site (with refs)	description	dates	location coordinates (in decimal degrees)	*n*	PM_2.5_ (μg m^–3^)	PM_2.5_ OC (μg m^–3^)	PM_2.5_ EC (μg m^–3^)	TPB (pg m^–3^)
Atlanta, Georgia^39^	urban	23–27 Aug, 2015	33.778944, −84.396167	4	9.1–14	3.3–5.2	0.26–0.32	3.9–30
Atlanta, Georgia^39^	urban	19–22 Jan, 2016	33.778944, −84.396167	4	6.4–14	1.5–4.9	0.16–0.58	19–64
Centreville, Alabama^41^	rural	12–14 July, 2013	32.902, −87.250	4	3.6–14	2.0–4.5	0.23–0.40	2.9–16
Iowa City, Iowa^38^	peri-urban	14–17 Nov, 2015	41.6647, −91.5845	4	NM^a^	1.2–9.6	0.05–0.81	2.6–42
Iowa City, Iowa	peri-urban	Oct–Nov, 2020	41.6647, −91.5845	10	NM^a^	1.0–3.1	0.08–0.39	21–70
Houston, Texas^40^	urban	18–20 May, 2015	29.733943, −95.257684	3	11–20	2.8–3.5	0.93–1.2	9.2–28
Bhola, Bangladesh^42^	background	April–July, 2013	22.166944, 90.750000	4	32–70	9.3–20^b^	2.9–6.3^b^	ND^c^
Dhaka, Bangladesh^43^	urban	Feb–April, 2013	23.72839, 90.39819	3	48–232	12–60^b^	4–21^b^	220–3500

a Not measured.

b Estimated by mean OC and EC mass fractions of PM_2.5_ observed previously (see refs).

c Not detected.

**Figure 3:**
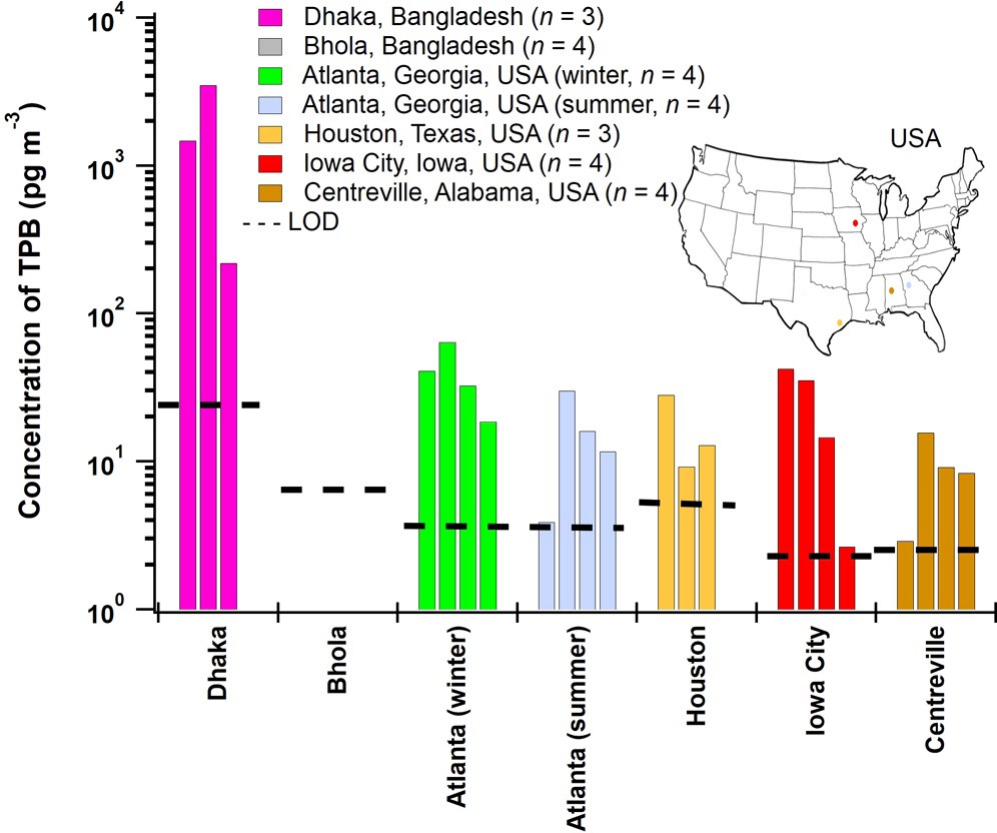
Concentrations of TPB (pg m^–3^) in ambient PM_2.5_ are shown on a logarithmic scale. The dashed line provides the limit of detection for each site. TPB was below the limit of detection in all samples from Bhola and one sample from Atlanta (summer). Limits of detection (LOD) in pg m^–3^ were determined by dividing the LOD (Table 1) by the mean volume of air analyzed for each site. Additional measurements for Iowa City are shown in Figure S4.

The observed concentrations of TPB spanned more than 3 orders of magnitude (Figure 3). TPB concentrations observed across the four sites in the USA spanned 2.9–25 pg m^–3^ and the urban site in Dhaka, Bangladesh, ranged 220–3500 pg m^–3^. The highest concentration was 3500 pg m^–3^ on February 3, 2013, in Dhaka, while the lowest quantifiable TPB concentration was 2.9 pg m^–3^ during the daytime of July 12, 2013, in Centreville, Alabama.

For comparison of TPB concentrations within a site over time, TPB concentrations were normalized to PM_2.5_ organic carbon (OC) to account for temporal differences in PM_2.5_ OC. The comparison of TPB concentrations in Atlanta across summer 2015 (4.0 ± 3.6 pg μg^–1^, mean ± standard deviation, *n* = 4) and winter 2016 (13.0 ± 0.9 pg μg^–1^, *n* = 4) indicates a significantly higher relative impact of plastic burning on PM_2.5_ during wintertime (*p* = 0.003). Comparing the OC-normalized TPB concentrations in Iowa City from November 2015 (9.1 ± 8.1 pg μg^–1^, *n* = 4) to those in October–November 2020 (21.1 ± 6.0 pg μg^–1^, *n* = 10, Figure S4) indicates a statistically significant increase in TPB relative to OC over this five-year time span (*p* = 0.01). Moreover, the TPB-to-OC ratios in ambient air were variable, with coefficients of variation of 0.9 in Iowa City (2015) and Atlanta (summer); 0.7 in Houston; 0.6 in Centreville; 0.3 for Iowa City (2020), and 0.1 in Atlanta (winter). This variability suggests day-to-day variability in the plastic burning impact on OC, reflecting intermittent sources that may be local or regional in nature.

The observed TPB levels in Dhaka were similar in magnitude to prior studies in South Asia, including Kathmandu, Nepal (250–2900 pg m^–3^);^22^ Lumbini, Nepal (570–4000 pg m^–3^);^23^ Raipur, India (80–15 400 pg m^–3^);^48^ Chennai, India (300–5000);^32^ Kuala Lumpur, Malaysia (urban average 2100 pg m^–3^)^49^ as well as other urban sites in Mexico City, Mexico (2000–4000 pg m^–3^);^29^ Bucharest, Romania (2700–3600 pg m^–3^);^10^ and Wadowice, Poland (260–2600 pg m^–3^).^33^ Notably, plastic and/or garbage burning was identified as an important source of PM_2.5_ in many of these studies, particularly those in South Asia, based upon the observed levels of TPB and, in some cases, other plastic burning tracers. In the Kathmandu Valley, garbage burning was estimated to contribute 18% of PM_2.5_ OC (equivalent to 3.2 μgC m^–3^) during April 2015 using molecular marker-driven chemical mass balance modeling, placing garbage burning among the major anthropogenic sources of open biomass burning (17%) and gasoline and diesel engines (18%).^22^ A similar impact of waste burning was reported in Lumbini, Nepal, in December 2017 at an average of 5% of PM_2.5_ OC (corresponding to an average of 2.8 μgC m^–3^).^23^ In India, plastic burning was among the five major PM_2.5_ sources assessed by PMF, contributing 13.4% of PM.^24^ Based on the TPB and PM levels in Dhaka, it is expected that plastic and waste burning has a significant air quality impact.

In contrast, the observed TPB levels at four sites in the USA (2.6–70 pg m^–3^; Table 2) were approximately 100 times lower than those observed in Dhaka, Bangladesh. The TPB concentrations observed in the USA were similar to those observed in Okinawa, Japan (7–88 pg m^–3^),^31^ and were slightly elevated in comparison to the mountain top site at Mt. Bachelor in Oregon (where TPB was detected in 26% of samples up to 26 pg m^–3^).^34^ Few measurements of TPB for urban areas in the USA have been documented, aside from nondetects in PM samples from Los Angeles, California, and Corvallis, Oregon.^2^ Taken together, these data demonstrate a chemical fingerprint of plastic burning at urban and rural sites in the USA.

### 3.3 Potential impacts of Plastic burning on PM_2.5_ in the USA and Bangladesh

In an effort to assess the potential impact of plastic burning on PM_2.5_ at these study sites, the potential impact of plastic burning on ambient PM_2.5_ mass concentrations (μg m^–3^) at the four study sites in the USA and in Dhaka, Bangladesh, was roughly estimated as the ratio of the TPB concentration (*C*_TPB_, ng m^–3^) to the TPB mass fraction of PM at the source of plastic combustion (*C*_TPB_*C*_PM_^–1^, ng μg^–1^) following eq 1
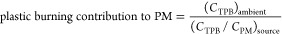
1This calculation assumes that the 1,3,5-isomer of TPB is unique to plastic burning and is conserved from the source to the receptor. The specificity of 1,3,5-TPB to plastic burning is supported by numerous studies on the combustion of plastic or waste materials containing plastic (Table S2) and the absence of TPB in combustion emissions from other when plastic is not present.^2,9,10,50^ TPB is predominant in the particle phase in the atmosphere,^31^ with >90% in the particle phase at elevated temperatures near its emission source.^50^ Any loss of TPB (i.e., due to photolysis, multiphase reactions, or oxidation) would underestimate the plastic burning impact on ambient particulate matter. Additionally, this estimation accounts only for plastic combustion and does not include estimates of mass contributions of co-fired materials.

The magnitude of the plastic burning source contribution depends upon the TPB mass fraction at the source. The lower limit of the plastic burning contribution to PM_2.5_ corresponds to the source profile with the maximum TPB mass fraction in PM, which was observed for polystyrene combustion in a residential stove (800 μg g^–1^) by Hoffer et al.^10^ The TPB mass fractions reported in the literature (Table S2) vary with the type of plastic combusted, with the highest mass fractions of TPB resulting from combustion of plastics with aromatic rings in their structures.^10^ In the case of polyethylene combustion (PE), TPB mass fractions in PM range from below detection limits to 63 μg g^–1^ indicating variability with the source material and combustion conditions.^2,9,10^ The median estimate of the plastic burning contribution to PM_2.5_ was estimated using a TPB mass fraction of 100 μg g^–1^. This TPB mass fraction was observed for polyethylene terephthalate (PET) plastic combustion in a residential stove^10^ and is the median of the five highest TPB mass fractions reported in the literature (Table S2). Mass fraction values below 8 μg g^–1^ (or 1% of the maximum value) were excluded from the median determination because these types of plastic burning are unlikely to contribute appreciably to ambient TPB concentrations.

Estimated plastic burning contributions to PM_2.5_ mass in the USA were <1 μg m^–3^ ranging from lower estimates of tenths of a percent to median estimates of a few percent (Table 3). These results demonstrate a consistent but relatively small relative impact of plastic burning on ambient PM_2.5_ mass at these study sites. Using background levels of TPB in the USA, the background contribution of plastic burning to PM_2.5_ mass and organic carbon was estimated. TPB was quantified in all of the 29 samples from the four study sites in the USA, having a minimum concentration of approximately 3 pg m^–3^ at the Iowa City, Atlanta (summer), and Centreville sites. This TPB concentration is similar to the lowest detectable concentrations of TPB at the mountain top site at Mt. Bachelor, Oregon,^34^ supporting that it represents background levels. Taking this as the background level and dividing by the TPB mass fractions in PM of 800 and 100 μg g^–1^ described above, the lower and median estimates of the plastic burning background contributions to PM_2.5_ mass were 0.004 and 0.03 μg m^–3^, respectively. TPB levels were elevated at least three times greater than this background level in 26 of 29 samples analyzed from the USA. Such elevations imply local and/or regional sources of TPB and plastic burning.

**Table 3:** Estimates of Plastic Burning Contributions to PM_2.5_ Mass at Four Sites in the USA and in Dhaka, Bangladesh^a^

			lower estimate		median estimate	
site	dates of Study	*n*	PM_2.5_ mass (μg m^–3^)	PM_2.5_ mass (%)	PM_2.5_ mass (μg m^–3^)	PM_2.5_ mass (%)
Atlanta, Georgia	24–27 Aug, 2015	4	0.005–0.05	0.04–0.4	0.04–0.3	0.3–3
Atlanta, Georgia	19–22 Jan, 2016	4	0.02–0.08	0.3–0.8	0.2–0.6	2–7
Houston, Texas	18–20 May, 2015	3	0.01–0.04	0.1–0.2	0.09–0.3	∼1
Iowa City, Iowa	14–17 Nov, 2015	4	0.003–0.05	NA	0.03–0.4	NA
Iowa City, Iowa	16 Oct–12 Nov, 2020	10	0.03–0.09	NA	0.3–0.7	NA
Centreville, Alabama	12–14 July, 2013	4	0.004–0.02	0.03–0.3	0.03–0.2	0.3–2
Dhaka, Bangladesh	Feb–April, 2013	3	0.3–4	0.6–2	2–35	5–15

a Lower and median values were calculated using TPB-to-PM emission ratios for polystyrene (Hoffer et al.^10^) and the median of select literature values (Table S2), respectively. Contributions to PM_2.5_ OC are reported in Table S3.

The absolute and relative impact of plastic burning on PM_2.5_ in Dhaka was estimated to be in the range of a few percent and up to 15% (Table 3). Although based on only three samples, these calculations suggest a potentially significant impact of plastic burning on ambient PM_2.5_ in Dhaka. Plastic burning was estimated to have a similar impact in Delhi, India (13.4% of PM),^24^ supporting a significant air quality impact of this source in the region. Considering that plastic is likely to be co-fired with other waste materials, the overall impact of garbage burning on PM_2.5_ maybe 2–8 times greater, following that TPB mass fractions observed in emissions from the open burning of mixed waste burning (Table S2). The larger estimated impact of plastic burning in Bangladesh compared to the four sites in the USA follows trends in the estimated quantity of waste burned in each nation, with an estimated 2.9 million metric tons of waste burned in the USA (primarily at residences) and 14.3 million metric tons burned in Bangladesh (including residences and dump sites).^1^

### 3.4 Plastic Burning Impacts on PM_2.5_ Organic Carbon (OC) in the USA and Bangladesh

The potential impact of plastic burning on PM_2.5_ organic carbon (OC) was estimated from ambient TPB concentrations and the TPB mass fraction in particle-phase OC. Such estimates are useful in assessing the relative impact of plastic burning when OC is measured but PM is not (i.e., the Iowa City site) and when source apportionment is performed on OC. Because OC was not measured in most emissions tests (Table S2), OC was assumed to account for 50% of the PM mass emitted from burning plastic, which allowed TPB mass fractions in PM to be converted to TPB mass fractions in PM OC. This value is in the middle of the range of PM_2.5_ OC mass fractions observed for household waste burning in China (40%),^6^ garbage fires surrounding Mexico City, Mexico (51–58%),^8^ and open burning of garbage in Nepal (median 60%).^9^ The lower limit of the plastic burning contribution to OC was estimated from a TPB-to-OC mass fraction of 1600 μg g^–1^ (which is calculated from emissions data for polystyrene combustion in a residential stove^10^) and a median value of 200 μg g^–1^ (which corresponds to combustion of polyethylene terephthalate)^10^ and corresponds to the median value calculated in Table S2). Higher estimates of plastic burning contributions to PM_2.5_ OC would result from the use of source profiles with lower TPB mass fractions of OC, which occurs for other types of plastic and household wastes,^10^ co-fired plastic and wood,^50^ and open burning of plastics with other waste materials.^2^

The impact of plastic burning on PM_2.5_ OC at the four sites in the USA is relatively small (Table S3). For example, in Houston, plastic burning contributions to PM_2.5_ OC are a few tenths of a percent for the lower estimate and a few percent for the median estimate; this estimated source contribution is small in comparison to the PM_2.5_ sources resolved by molecular marker-based positive matrix factorization (PMF), including diesel engines (12% of OC), gasoline engines (24%), non-tailpipe vehicle emissions (11%), ship emissions (2%), biomass burning (11%), and secondary organic aerosol (SOA) (39%).^40^ In Centreville, a similarly small impact of plastic burning on PM_2.5_ OC was detected, especially in comparison to the major sources of aerosol estimated by molecular marker-driven chemical mass balance modeling, molecular marker-based PMF, and aerosol mass spectrometry (AMS)-driven PMF: biomass burning (5–10% OC), vehicle emissions (5–8%), and SOA (>60%).^53^ Taken together, these data demonstrate that plastic burning is expected to be a relatively minor source of PM_2.5_ OC in comparison to other anthropogenic sources and SOA at the four study sites in the USA.

In Dhaka, the absolute and relative impact of plastic burning on PM_2.5_ OC was greater (Table S3). The estimated contributions of plastic burning to PM_2.5_ in Dhaka were similar to prior studies in the Kathmandu Valley in April 2015, where garbage burning was estimated to contribute 18% of PM_2.5_ OC (equivalent to 3.2 μgC m^–3^),^22^ and in Lumbini, Nepal in December 2017, where garbage burning contributed an average of 5% of PM_2.5_ OC (equivalent to 2.8 μgC m^–3^).^23^ When considering either PM_2.5_ OC or mass, plastic burning is expected to have a significant impact on air quality in Dhaka.

## 4 Conclusions

Herein, we demonstrate the facile integration of TPB measurement into two common methods for organic speciation of atmospheric PM_2.5_: solvent-extraction GCMS and thermal-desorption GCMS. We reaffirm the recommendation of Simoneit^27^ to integrate TPB measurements into routine aerosol analysis, as it behaves similarly to PAH in its molecular weight range that are commonly measured and provides new insight into the occurrence of plastic and waste burning. Additional ambient measurements of TPB are needed to understand the air quality and health impacts of plastic combustion. Assessments of waste burning more broadly should include molecular tracers associated with other types of plastic and waste burning,^2,10^ to capture the diverse range of materials that are combusted. Concurrently, further studies on source emissions are needed to represent different waste compositions and burning conditions that are expected to vary regionally.

The measurements presented herein provide new insight into the levels of TPB in the USA and provide constraints on the potential impact of plastic burning on PM_2.5_ organic carbon. Additionally, these results demonstrate a much larger impact of plastic burning on PM_2.5_ in Dhaka, Bangladesh. These findings indicate the potential for chronic exposure to plastic and waste burning emissions. While this work has been concerned with measurements of ambient PM_2.5_, the greatest human exposures are likely to occur near waste burning points or area sources. The health impacts associated with such exposures are likely significant following the established toxicity of plastic burning emissions.

## Supporting Information Available

Optimization of TD-GCMS inlet conditions; time events and temperatures for TD-GCMS (Figure S1); optimization of inlet temperatures and desorption time (Figure S2), mass spectra of TPB collected by solvent-extraction and thermal-desorption GCMS methods (Figure S3); additional measurements of TPB in Iowa City, Iowa from 2020 (Figure S4); summary of GCMS conditions (Table S1); summary of TPB and PM emissions from burning plastic and mixed waste (Table S2); and estimates of plastic burning contributions to PM_2.5_ organic carbon (OC) at four sites in the USA and Dhaka, Bangladesh (PDF)

## Abbreviations

PM: particulate matter
OC: organic carbon
TPB: 1,3,5-triphenylbenzene
GCMS: gas chromatography mass spectrometry
TD: thermal desorption
QFF: quartz fiber filter
PAH: polycyclic aromatic hydrocarbon
