# Supplementary material for: Plastic Burning Impacts on Atmospheric Fine Particulate Matter at Urban and Rural Sites in the USA and Bangladesh
Source: ACS Environ Au. 2022 Jun 9;2(5):409–17. doi: 10.1021/acsenvironau.1c00054 (PMC9502013; doi:10.1021/acsenvironau.1c00054)
Supplement: Supplementary file 1 — vg1c00054_si_001.pdf [file vg1c00054_si_001.pdf]

**SUPPORTING INFORMATION**

# Plastic burning impacts on atmospheric fine particulate matter at urban and rural sites in the United States and Bangladesh

Md. Robiul Islam[1], Josie Welker[1], Abdus Salam[2], Elizabeth A. Stone[1,3]*

[1]University of Iowa, Department of Chemistry, Iowa City, IA, 52242 USA
[2]University of Dhaka, Department of Chemistry, Dhaka 1000, Bangladesh
[3]Department of Chemical and Biochemical Engineering, University of Iowa, Iowa City, IA, 52242 USA
*Corresponding author e-mail: betsy-stone@uiowa.edu

Supplemental Text 1.

Thermal desorption (TD) is a method of introducing ambient PM collected on substrates into a gas chromatograph (GC).[1-4] In this technique, a small strip of aerosol containing filter is packed into a GC inlet liner. This liner then loaded into GC inlet and heated to 275-300 °C to desorb the volatile and semi-volatile compounds. During the desorption, the column temperature is maintained at 30 °C to concentrate and focus the analytes at the GC column head. Then the GC column temperature is gradually raised to facilitate the chromatographic separation.

The optimization of the GC inlet temperature for thermal desorption is a balance of fully desorbing analytes without thermal decomposition. Standard solutions containing TPB with benzo(a)anthracene-D12 as an internal standard were spiked on blank filters and analyzed at four different temperatures: 200, 250, 275, and 300 °C. The time required for the injector to reach 200, 250, 275, and 300 °C was 6.5, 9.0, 11.0, and 12.5 min respectively. For all experiments, the GC oven temperature ramp was initiated 14 min after the start of thermal desorption to maintain a constant total thermal desorption time.

The responses of TPB and benzo(a)anthracene-d12 at four thermal desorption temperatures were normalized by their responses at 300 °C and are plotted in Figure S2a. The responses of both compounds increased as the temperature increased from 200 to 275 °C. Increasing inlet temperature further to 300 °C increased the response for benzo(a)anthracene by 9%, however it also decreased the response for TPB by 2%. These results indicated that temperatures of 250 °C or lower are not sufficient for complete thermal desorption of TPB. The temperature of 275 °C was selected, because further increase in temperature did not increase the analyte response. This same desorption temperature was determined to be optimum for the analysis of semi-volatile compounds including polycyclic aromatic hydrocarbons (PAHs), alkanes, and phthalates in prior studies.[5, 6]

The optimum duration for thermal desorption is the minimum time that is required for complete desorption of analytes from the filter substrate and their subsequent deposition at the GC column head. After the injector reached to 275 °C, four time intervals, 1, 2, 3, and 5 min, were allowed to elapse before the GC temperature program was started. The responses of TPB and benzo(a)anthracene-d12 at these four elapsed times are plotted in S2b. Increasing the elapsed desorption time from 1 min to 2 min, increased the responses of both analyte by 5-8% indicating a better desorption. Further increase in elapsed time to 3 min increased the response of TPB by 11-14%, however decreased the response of benzo(a)anthracene by 3-5% compared to their responses at 1 min elapsed time (Figure 2.4b). A subsequent increase in desorption time did not alter the responses for any of the analytes. Based on

these observations, the 2 min elapsed time was selected as optimum desorption time as it was the minimum required time with maximum desorption efficiency for both analytes. The same elapsed time was used in the analysis of PAHs, alkanes, and phthalates in prior studies.[6, 7]

**Table S1**. Summary of gas chromatography mass spectrometry (GCMS) operating conditions for the solvent extraction and thermal desorption methods.

| **Component** | **Operating Conditions** | **Solvent extraction GCMS** | **Thermal Desorption-GCMS** (with direct sample introduction) |
|---|---|---|---|
| **Injector** | Injection volume (µL) | 2 | - |
| | Solvent wash draw speed (µL/min) | 300 | - |
| **Inlet** | Carrier gas | Helium (99.999%) | Helium (99.999%) |
| | Mode | Splitless | Splitless |
| | Heater (°C) | 300 | (See Figure S1) |
| | Pressure (psi) | 8.5204 | 11.084 |
| | Total flow (mL/min) | 70.2 | 70.681 |
| | Septum purge flow (mL/min) | 3 | 3 |
| | Purge flow to split vent (mL/min) | 66.2 at 1 min | 66.2 at 13 min |
| **GC** | Column | HP-5MS (Agilent, 30 m x 0.25 mm x 0.25 µm) | HP-5MS (Agilent, 30 m x 0.25 mm x 0.25 µm) |
| | Oven Program | Initial 65 °C; hold 10 min, ramp at 10 °C/min to 300 °C, hold 25 min | Initial 30 °C, hold 13 min, ramp at 10 °C/min, to 120 °C, ramp at 8 °C/min to 310 °C, hold 13 min |
| **GC Transfer Line** | Setpoint (°C) | 300 | 300 |
| **MS Data Acquisition** | Solvent delay (min) | 11 | 16 |
| | Mass range (m/z) | 50-500 | 50-500 |
| | Mode | Scan | Scan |
| | MS source (°C) | 230 | 230 |
| | MS Quad (°C) | 150 | 150 |

**Table S2.** Summary of PM and TPB emissions data for the combustion of I) plastic and II) mixed waste containing plastic.

| Combusted materials | Burning conditions | Location of testing | PM size | $EF_{PM}$ (mg $kg^{-1}$) | | | TPB/PM (ng $mg^{-1}$) | TPB/OC (ng $mgOC^{-1}$) | Reference |
|---|---|---|---|---|---|---|---|---|---|
| **I. Plastic** | | | | | | | | | |
| Polystyrene (PS) | stove | Romania | $PM_{10}$ | 53 | ± | 15 | 800 | nm | Hoffer et al. 2021[8] |
| Polyethylene (PE), polyethylene terephthalate (PET), polyvinyl chloride (PVC), PS | open burning | Chile | TSP | | nm[A] | | 208 | nm | Simoneit et al. 2005[9] |
| PET | stove | Romania | $PM_{10}$ | 11 | ± | 1.6 | 100 | nm | Hoffer et al. 2021[8] |
| PE (new bags) | open burning | Chile | TSP | | nm | | 63 | nm | Simoneit et al. 2005[9] |
| Acrylonitrile-butadiene-styrene (ABS) | stove | Romania | $PM_{10}$ | 82 | ± | 27 | 24 | nm | Hoffer et al. 2021[8] |
| mixed plastics (predominantly PE) | open burning | Nepal | $PM_{2.5}$ | 84 | ± | 13 | 7 | 11 | Jayarathne et al. 2018[10] |
| PVC | stove | Romania | $PM_{10}$ | 35 | ± | 10 | 6.2 | nm | Hoffer et al. 2021[8] |
| Polypropylene (PP) | stove | Romania | $PM_{10}$ | 33 | ± | 18 | 0.5 | nm | Hoffer et al. 2021[8] |
| PE (new bags) | open burning | USA | TSP | | nm | | 0.2 | nm | Simoneit et al. 2005[9] |
| PE | stove | Romania | $PM_{10}$ | 18 | ± | 7 | bdl[B] | nm | Hoffer et al. 2021[8] |
| **median of plastics[C]** | | | | | | | **100** | **200[D]** | |
| **II. Mixed waste containing plastic** | | | | | | | | | |
| landfill waste, 3.2% plastic (PE 17.3%, PET 29.7%, PVC 39.3%, PS 2.9%, unidentified 10.8%) | open burning | Chile | TSP | | nm | | 57 | nm | Simoneit et al. 2005[9] |
| rags made of a mixture of cotton, polyester and polyamide fabrics | stove | Romania | $PM_{10}$ | 8.7 | ± | 0.22 | 15 | nm | Hoffer et al. 2021[8] |
| paper, including colorful glossy-coated and uncoated | stove | Romania | $PM_{10}$ | 2.2 | ± | 0.11 | 75 | nm | Hoffer et al. 2021[8] |
| furniture made of low density fiber board, including laminated coating and plastic borders | stove | Romania | $PM_{10}$ | 3.2 | ± | 1.3 | 23 | nm | Hoffer et al. 2021[8] |
| mixed waste (damp food waste, paper, plastic bags, cloth, diapers, and rubber shoes) | open burning | Nepal | $PM_{2.5}$ | 125 | ± | 23 | 15 | 25 | Jayarathne et al. 2018[10] |
| mixed waste (damp food waste, paper, plastic bags, cloth, diapers, and rubber shoes) | open burning | Nepal | $PM_{2.5}$ | 82 | ± | 13 | 12 | 20 | Jayarathne et al. 2018[10] |
| mixed waste (dry domestic waste, containing cardboard and chip bags) | open burning | Nepal | $PM_{2.5}$ | 7 | ± | 1 | 51 | 45 | Jayarathne et al. 2018[10] |
| foil wrappers (damp) | open burning | Nepal | $PM_{2.5}$ | 50 | ± | 9 | 5 | 7 | Jayarathne et al. 2018[10] |
| PET (bottles) co-combusted with beech wood (7:93% by weight) | boiler | Czech Republic | TSP | 1.8 | ± | 0.3 | 13 | nm | Tomsej et al. 2018[11] |
| PE (new bags) co-combusted with beech wood (7:93% by weight) | boiler | Czech Republic | TSP | 1.7 | ± | 0.2 | 4 | nm | Tomsej et al. 2018[11] |
| PET (bottles) co-combusted with beech wood (reduced output, 7:93% by weight) | boiler | Czech Republic | TSP | 5.8 | ± | 0.7 | 13 | nm | Tomsej et al. 2018[11] |
| PE (new bags) co-combusted with beech wood (reduced output, 7:93% by weight) | boiler | Czech Republic | TSP | 7.5 | ± | 0.5 | 1 | nm | Tomsej et al. 2018[11] |

A) Not measured, B) below detection limit, C) Excludes values < 10, which are not expected to contribute appreciably to ambient TPB concentrations; D) Estimated by assuming that OC accounts for 50% of PM mass.

**Table S3.** Estimates of plastic burning contributions to $PM_{2.5}$ organic carbon (OC) at four sites in the USA and in Dhaka, Bangladesh. Lower and median values were calculated using TPB-to-PM emission ratios for polystyrene (Hoffer et al. 2021) and the median of select literature values (Table S2), respectively.

| Site | Dates of Study | *n* | Lower estimate | | Median estimate | |
|---|---|---|---|---|---|---|
| | | | $PM_{2.5}$ OC (μgC $m^{-3}$) | $PM_{2.5}$ OC (%) | $PM_{2.5}$ OC (μgC $m^{-3}$) | $PM_{2.5}$ OC (%) |
| Atlanta, Georgia, USA | 24-27 Aug 2015 | 4 | 0.002-0.02 | 0.06-0.6 | 0.02-0.2 | 0.5-5 |
| Atlanta, Georgia, USA | 19-22 Jan 2016 | 4 | 0.01-0.04 | 0.8-0.9 | 0.09-0.3 | 6-7 |
| Houston, Texas, USA | 18-20 May 2015 | 3 | 0.006-0.02 | 0.2-0.6 | 0.05-0.1 | 1-5 |
| Iowa City, Iowa, USA | 14-17 Nov 2015 | 4 | 0.002-0.03 | 0.1-1 | 0.07-0.2 | 1-10 |
| Iowa City, Iowa, USA | 16 Oct – 12 Nov 2020 | 10 | 0.02-0.04 | 1-2 | 0.1-0.4 | 6-17 |
| Centreville, Alabama, USA | 12-14 July 2013 | 4 | 0.002-0.01 | 0.04-0.3 | 0.01-0.08 | 0.4-2 |
| Dhaka, Bangladesh | Feb – April 2013 | 3 | 0.1-2 | 1-3 | 1-17 | 8-26 |

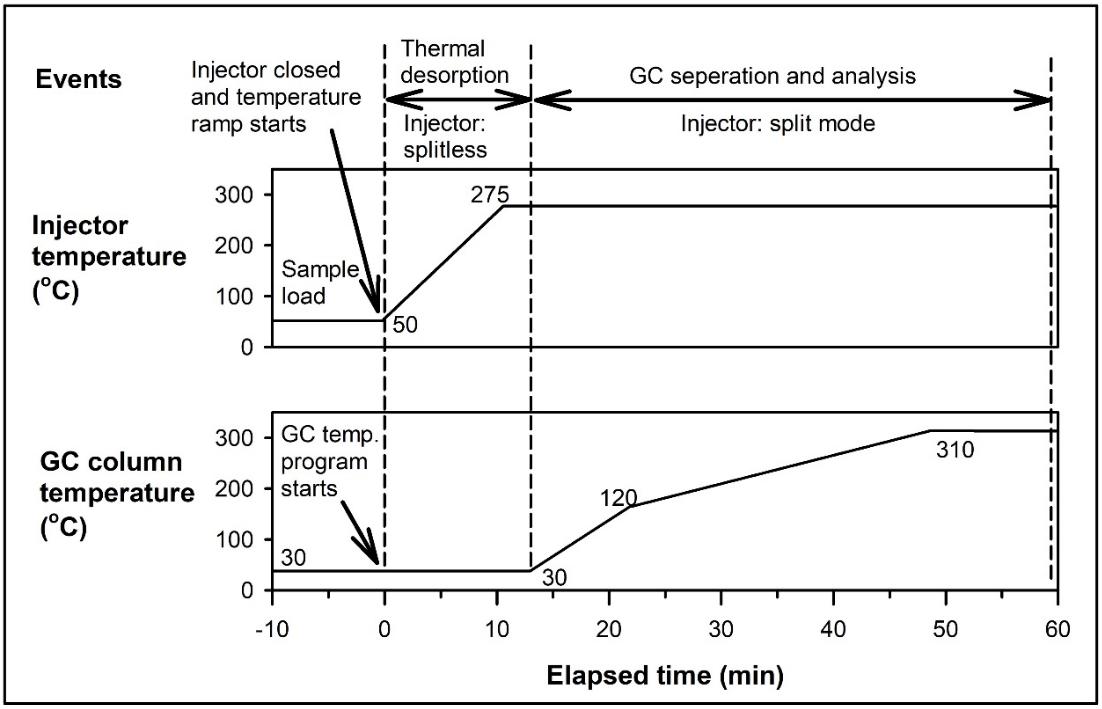


**Figure S1.** Time events and temperature programs for GC inlet and column oven during the TD-GCMS analysis.

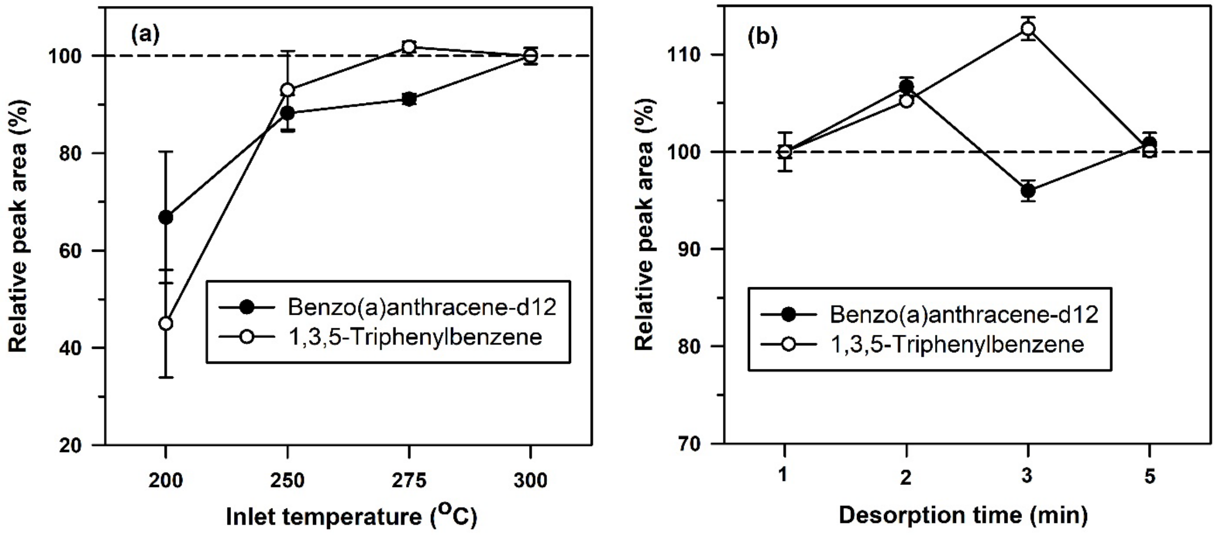


**Figure S2.** Optimization of inlet temperature and desorption time for the TD-GCMS analysis of TPB.

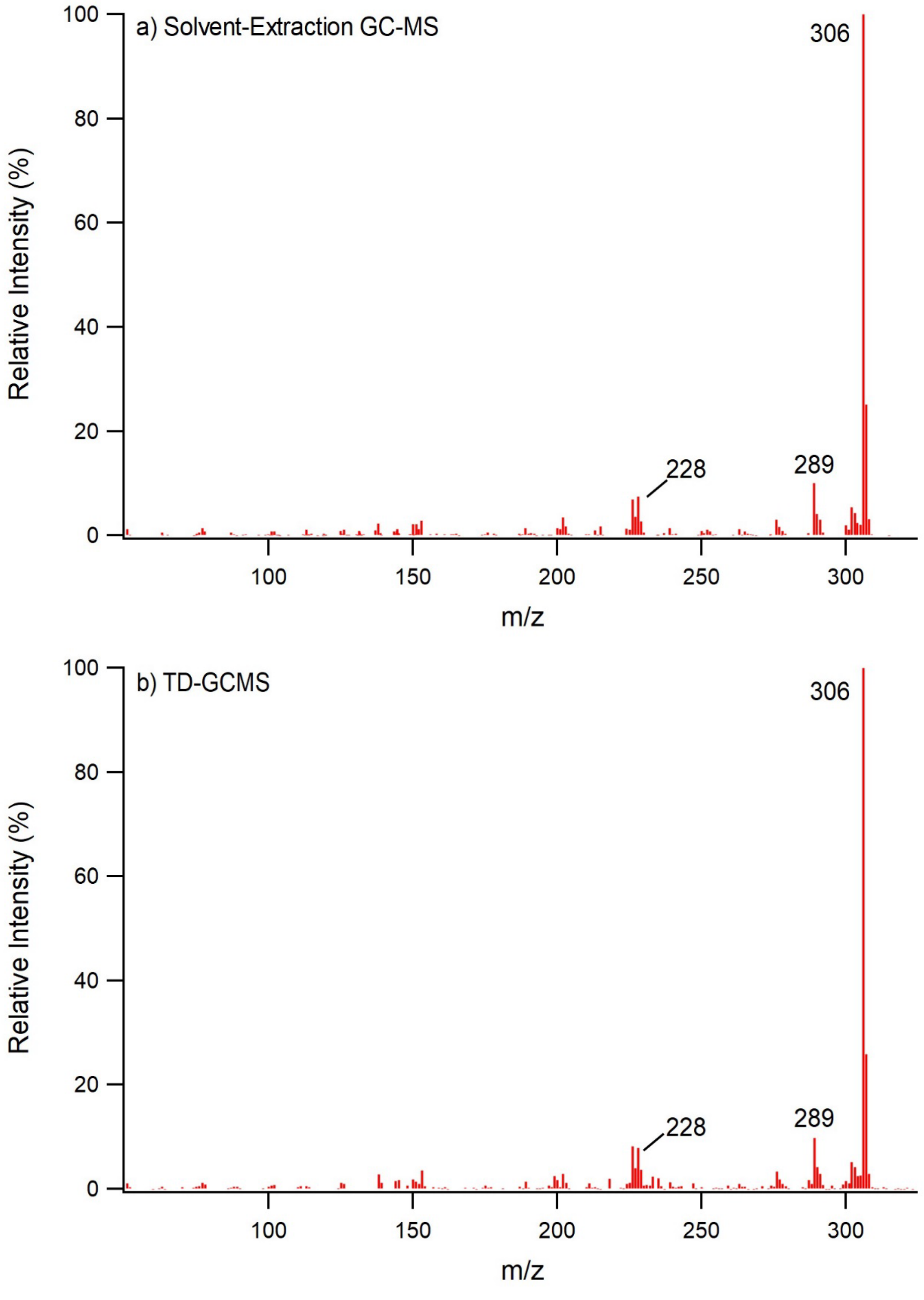


**Figure S3.** Mass spectra for 1,3,5-triphenylbenzene (TPB) collected under electron ionization at 70 eV using the solvent-extraction GCMS and thermal desorption-GCMS conditions outlined in Table S1. The three *m/z* marked were used to identify and quantify TPB.

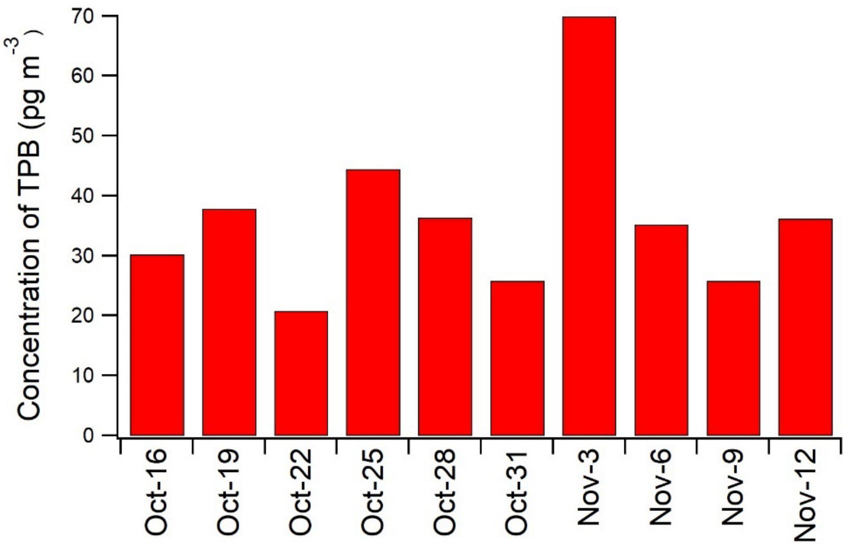


**Figure S4**: TPB concentrations measured in Iowa City, Iowa, USA in 2020.

## REFERENCES


1. Falkovich, A. H.; Rudich, Y., Analysis of semivolatile organic compounds in atmospheric aerosols by direct sample introduction thermal desorption GC/MS. *Environ. Sci. Technol.* **2001,** *35* (11), 2326-2333.

2. Hall, P. A.; Watson, A. F. R.; Garner, G. V.; Hall, K.; Smith, S.; Waterman, D.; Horsfield, B., An investigation of micro-scale sealed vessel thermal extraction-gas chromatography-mass spectrometry (MSSV-GC-MS) and micro-scale sealed vessel pyrolysis-gas chromatography-mass spectrometry applied to a standard reference material of an urban dust/organics. *Sci. Total Environ.* **1999,** *235* (1-3), 269-276.

3. Sigman, M. E.; Ma, C. Y., In-injection port thermal desorption for explosives trace evidence analysis. *Anal. Chem.* **1999,** *71* (19), 4119-4124.

4. Waterman, D.; Horsfield, B.; Leistner, F.; Hall, K.; Smith, S., Quantification of polycyclic aromatic hydrocarbons in the NIST standard reference material (SRM1649A) urban dust using thermal desorption GC/MS. *Anal. Chem.* **2000,** *72* (15), 3563-3567.

5. Ho, S. S. H.; Yu, J. Z., In-injection port thermal desorption and subsequent gas chromatography-mass spectrometric analysis of polycyclic aromatic hydrocarbons and n-alkanes in atmospheric aerosol samples. *J. Chromatogr. A* **2004,** *1059* (1-2), 121-129.

6. Ho, S. S. H.; Yu, J. Z.; Chow, J. C.; Zielinska, B.; Watson, J. G.; Sit, E. H. L.; Schauer, J. J., Evaluation of an in-injection port thermal desorption-gas chromatography/mass spectrometry method for analysis of non-polar organic compounds in ambient aerosol samples. *J. Chromatogr. A* **2008,** *1200* (2), 217-227.

7. Yu, J. Z.; Huang, X. H. H.; Ho, S. S. H.; Bian, Q. J., Nonpolar organic compounds in fine particles: quantification by thermal desorption-GC/MS and evidence for their significant oxidation in ambient aerosols in Hong Kong. *Analytical and Bioanalytical Chemistry* **2011,** *401* (10), 3125-3139.

8. Hoffer, A.; Gelencser, A.; Guyon, P.; Kiss, G.; Schmid, O.; Frank, G. P.; Artaxo, P.; Andreae, M. O., Optical properties of humic-like substances (HULIS) in biomass-burning aerosols. *Atmospheric Chemistry and Physics* **2006,** *6*, 3563-3570.

9. Simoneit, B. R. T.; Medeiros, P. M.; Didyk, B. M., Combustion products of plastics as indicators for refuse burning in the atmosphere. *Environmental Science & Technology* **2005,** *39* (18), 6961-6970.

10. Jayarathne, T.; Stockwell, C. E.; Bhave, P. V.; Praveen, P. S.; Rathnayake, C. M.; Islam, M. R.; Panday, A. K.; Adhikari, S.; Maharjan, R.; Goetz, J. D.; DeCarlo, P. F.; Saikawa, E.; Yokelson, R. J.; Stone, E. A., Nepal Ambient Monitoring and Source Testing Experiment (NAMaSTE): emissions of particulate matter from wood- and dung-fueled cooking fires, garbage and crop residue burning, brick kilns, and other sources. *Atmos. Chem. Phys.* **2018,** *18* (3), 2259-2286.

11. Tomsej, T.; Horak, J.; Tomsejova, S.; Krpec, K.; Klanova, J.; Dej, M.; Hopan, F., The impact of co-combustion of polyethylene plastics and wood in a small residential boiler on emissions of gaseous pollutants, particulate matter, PAHs and 1,3,5- triphenylbenzene. *Chemosphere* **2018,** *196*, 18-24.
